# The effectiveness of virtual reality for K-12 foreign language learning: a systematic review of recent randomized controlled trials

**DOI:** 10.3389/fpsyg.2025.1714481

**Published:** 2026-01-06

**Authors:** Lu Sun, Xiacheng Song

**Affiliations:** 1 Xi'an Fanyi University, Xi'an, China; 2 Faculty of Social Sciences and Humanities, Universiti Kebangsaan Malaysia, Selangor, Malaysia; 3 Institute of Visual Informatics, Universiti Kebangsaan Malaysia, Selangor, Malaysia

**Keywords:** educational technology, foreign language learning, K-12 education, systematic review, virtual reality

## Abstract

**Background:**

Despite the increasing adoption of immersive Virtual Reality (VR) in K−12 educational settings, there is a notable absence of systematic, high-quality experimental research evaluating its efficacy in facilitating foreign language acquisition.

**Methods:**

Following a systematic search of five databases that yielded 1,054 records, six randomized controlled trials (RCTs) met the inclusion criteria. Because of considerable heterogeneity, a narrative synthesis was conducted following the Synthesis Without Meta-analysis (SWiM) guideline, with findings structured into a primary contrast (VR vs. non-VR) and a secondary analysis (VR vs. VR designs).

**Results:**

The primary contrast analysis indicated that VR interventions generally had a positive effect compared to non-VR controls, particularly for vocabulary and listening. A notable finding was a consistent positive effect for VR in promoting long-term knowledge retention. The risk of bias evaluation indicated that each of the included studies was classified as presenting “some concerns”.

**Conclusion:**

Across a small and heterogeneous set of recent RCTs, immersive VR shows promising effects, especially for long-term retention. However, the evidence for immediate learning gains is inconclusive. A more critical finding is the profound heterogeneity and methodological concerns within the evidence base, which preclude any single, overarching conclusion about VR's effectiveness.

## Introduction

### The emergence of virtual reality in language education

In recent years, immersive technologies, particularly Virtual Reality (VR), have transitioned from novel concepts to viable pedagogical tools within mainstream education (Lee and Wu, 2023; Tschanz and Baerlocher, 2022). Virtual reality (VR) affords interactive three-dimensional (3D) environments that foster presence and embodied interaction (Figueroa et al., 2024). These affordances are particularly salient for second/foreign-language (L2) learning, where aligning classroom practice with authentic use remains a central challenge (Jauregi-Ondarra et al., 2021; Kolesnichenko, 2023). Traditional approaches often provide limited opportunities for contextualized, interactive use of the target language (Kolesnichenko, 2023). Virtual reality (VR) addresses this constraint by situating learners in realistic, task-relevant contexts that demand meaningful communication, consistent with theories of situated and experiential learning (Lee and Wu, 2023). By providing culturally natural settings and interactive manipulation of virtual entities or objects, VR also has the capacity to strengthen the motivation of the learner, reduce anxiety of communication, and yield greater cognitive elaboration of linguistic input (Baidya et al., n.d.; Jauregi-Ondarra et al., 2021).

### Why focus on K-12 L2 learning?

K-12 learners differ from adults in cognitive, socio-emotional, and motivational profiles, which may shape how immersive technologies influence attention, memory consolidation, and willingness to communicate (Jauregi-Ondarra et al., 2021). Schools also impose practical constraints limited session length, classroom management, and teacher mediation that can modulate VR's effectiveness relative to higher-education or informal learning settings (Cheng, 2012; Voordijk and Vahdatikhaki, 2020). A K-12–specific synthesis is therefore essential to inform curricular design, teacher training, and procurement decisions (Hite et al., 2019; Wilkinson et al., 2021).

Moreover, K−12 represents a critical developmental stage in which learners form foundational linguistic, cognitive, and socio-emotional skills (Arts et al., 2024; Pataquiva and Klimova, 2022; Weng et al., 2024). Early exposure to foreign-language learning has been shown to improve long-term proficiency, motivation, and intercultural competence. Therefore, understanding how immersive technologies support L2 learning at this stage is essential for educators and policymakers (Liu et al., 2023). This also underscores the timeliness and significance of conducting a systematic review specifically focused on K−12 L2 learning with immersive VR (Lee et al., 2023).

### What counts as “immersive VR” in this review

To avoid conflating distinct media, we conceptualize immersive VR primarily as head-mounted display (HMD)–based environments that enable first-person perspective, head-tracked presence, and goal-directed interaction with virtual elements (Buetler et al., 2022; Dahl et al., 2021; Tan et al., 2022). While display configurations can vary, studies were considered within scope when learners experienced embodied interaction and spatial presence consistent with this definition. Non-interactive panoramic/360° video without head tracking is not treated as immersive VR in this review (cf. Lee and Wu, 2023). While our definition centers on HMD-based environments, we also included studies using hybrid systems where HMDs were essential for interaction and presence, such as the 360-degree projection system combined with HMDs in the study by Chang et al. (2024). The potential influence of this technological variation is addressed in the limitations section.

### State of the evidence and the gap this review addresses

Empirical work on VR for L2 learning has proliferated, but the evidence remains fragmented: populations span grades and contexts; interventions differ in tasks, scaffolding, and exposure; outcomes vary across skills and timing (immediate vs. delayed); and reporting standards are uneven (Tai et al., 2022; Wu et al., 2021). Prior reviews often mixed higher-education with K-12 samples or combined immersive VR with non-immersive 3D/AR/360° media, limiting causal interpretability for school-age learners (Natale et al., 2020; Pellas et al., 2021). Moreover, delayed post-test outcomes critical for retention are inconsistently reported, and many studies have small samples or non-randomized designs that invite bias (Chen et al., 2023; Qiu et al., 2024). To provide policy- and practice-relevant guidance, there is a need for a focused, methodologically rigorous synthesis that (a) concentrates on K-12 learners, (b) uses a precise operationalization of immersive HMD-VR, (c) privileges randomized controlled trials (RCTs) to strengthen causal inference, and (d) distinguishes immediate from delayed outcomes across language domains.

Previous reviews of VR in education and language learning have generally taken a broad scope, often combining school-age learners with university students and mixing immersive HMD-based VR with less immersive 3D environments or 360° video, as well as non-randomized designs (Fransson et al., 2020; Wu et al., 2020). As a result, it is difficult to isolate what the highest-quality evidence suggests specifically for K−12 foreign language learning. By deliberately narrowing the focus to clearly defined immersive HMD-VR, randomized controlled trials, and school-aged learners, this review aims to offer a conservative but decision-relevant picture of what can currently be concluded from this emerging evidence base.

### The present study and contributions

This review systematically synthesizes recent studies evaluating immersive HMD-VR for K-12 foreign language learning, with an emphasis on randomized controlled trials (e.g., Wu et al., 2020); see also (Shen et al., 2023). It makes three contributions. First, it offers a K-12–specific account that isolates school-age evidence from adult and higher-education studies. Second, it applies a tight operationalization of immersive VR to avoid media conflation and to clarify what educators can expect from HMD-based interventions. Third, it disaggregates language outcomes by timing and domain, highlighting whether VR advantages are concentrated in immediate performance or long-term retention, and whether effects cluster in vocabulary, listening, or writing (Jwai'ed et al., 2024; Lai and Chen, 2023; Sahinler, 2023).

Accordingly, we examine the following research questions (RQs):

RQ1: Among recent RCTs focusing on K-12 learners, what is the effect of immersive VR, relative to non-VR control conditions, on L2 learning outcomes?
RQ2: How does this effect vary across distinct language domains (e.g., vocabulary, listening)?
RQ3: Does immersive VR improve delayed post-test performance, indicating stronger retention?
RQ4: How do different VR design features (e.g., level of immersion, pedagogical approach) compare in their effects on K-12 L2 learning outcomes?

*Scope note*. To maximize causal interpretability and align with school decision-making needs, this review focuses exclusively on RCTs (Alfadil, 2020; Chen et al., 2023; Qiu et al., 2024).

## Methods

This systematic review protocol was preregistered with the Open Science Framework (OSF) and is available at https://osf.io/wdx4f.

A systematic search was conducted across five electronic databases: Web of Science, Scopus, IEEE Xplore, ACM Digital Library, and ERIC. The search was conducted on September 18, 2025, and aimed to identify all relevant studies. In addition, we screened the reference lists of recent systematic reviews on VR and language education and searched for related terms such as “spherical video–based VR” and “social VR” to minimize the risk of missing eligible RCTs that used alternative terminology. No language or publication date restrictions were initially applied. The search query combined keywords related to three core concepts: Virtual Reality (e.g., “virtual reality,” “VR,” “immersive”), the K-12 population (e.g., “K-12,” “elementary,” “high school,” “children,” “adolescent”), and foreign language learning (e.g., “language learning,” “second language,” “foreign language,” “L2”). Search terms within each concept were combined using the ‘OR' operator, and the three concepts were then combined using the ‘AND' operator.

To improve transparency and reproducibility, we adapted this generic search template to the syntax of each database. In Web of Science, we searched topic fields (TS) using the three concept blocks combined with Boolean operators, whereas in Scopus, ERIC, IEEE Xplore, and ACM Digital Library we searched titles, abstracts, and keywords using equivalent terms. All search strings were constructed in English, but records with non-English full texts were retained during screening when an English title or abstract was available.

## Inclusion and exclusion criteria

Studies were included in this review if they met the following criteria (Moher et al., n.d.):

- Population: Focused on K-12 students (approximately 5–18 years old).
- Intervention: Employed immersive Virtual Reality (VR) tools such as head-mounted displays as the central instructional approach for second or foreign language acquisition.
- Outcomes: Reported quantifiable language learning outcomes (e.g., vocabulary acquisition, listening comprehension, writing skills).
- Study design: To maximize causal interpretability and align with the review's objective of evaluating effectiveness, only randomized controlled trials (RCTs) were included. Quasi-experimental, non-randomized, and single-group pre-test/post-test designs were systematically excluded due to their higher inherent risk of bias.

Exclusion criteria included non-empirical studies (e.g., opinion papers, descriptive reports), studies targeting university students or adults, interventions using non-immersive technology (e.g., 360° videos without interactivity, mobile apps), and studies where K-12 student data could not be separated from other populations.

### Study selection

**Figure 1:**
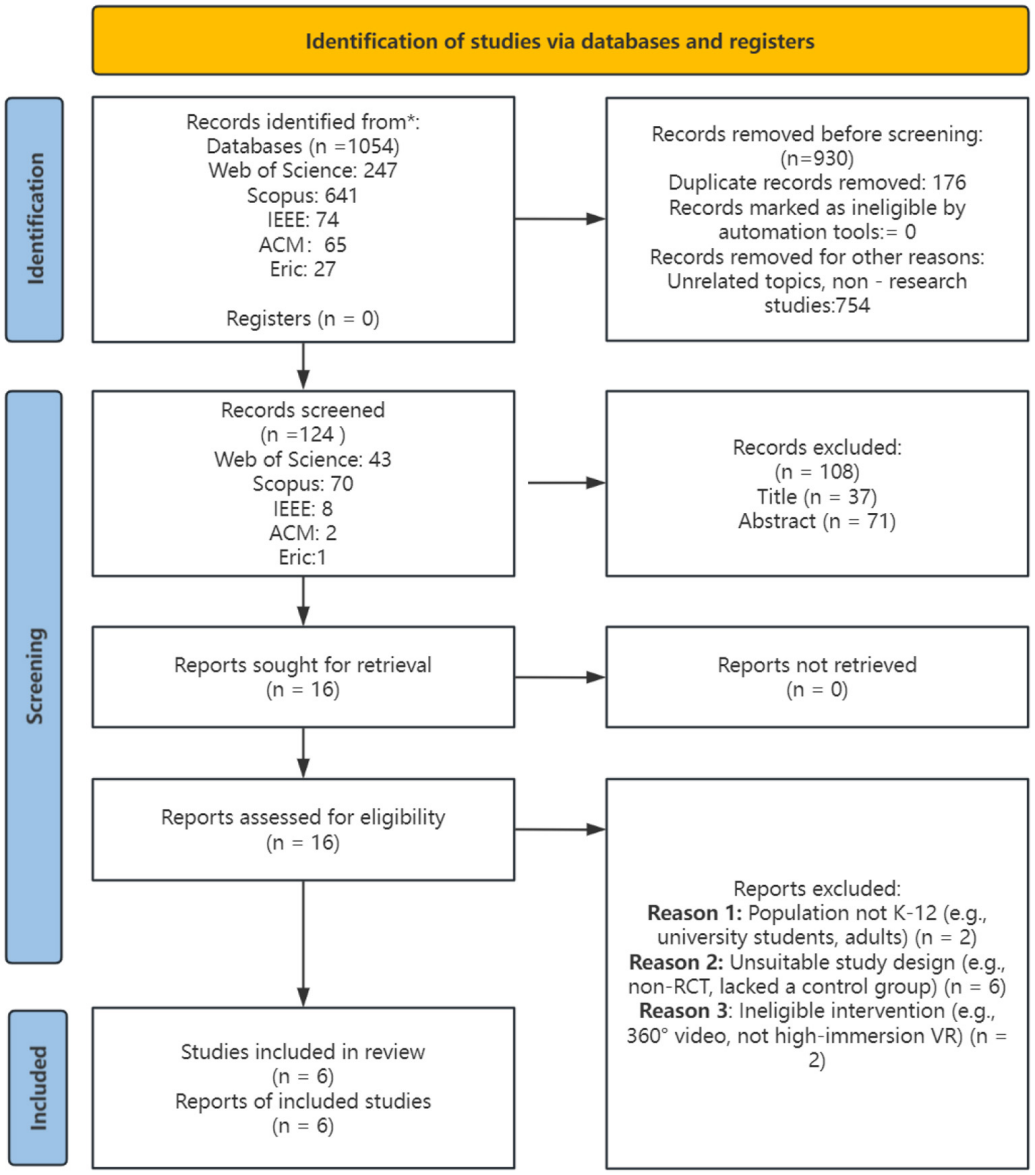
PRISMA flow diagram.

The study selection process was conducted by two independent reviewers (Author X and Author Y). Titles and abstracts were screened first, followed by a full-text assessment against the inclusion criteria. Any disagreements were resolved through discussion or, if necessary, consultation with a third reviewer (Author Z). An overview of the study screening procedure is presented (Page et al., 2021) (Figure 1). The initial database query yielded 1,054 entries. Following the exclusion of 930 records comprising 176 duplicates and 754 items removed due to irrelevance or non-academic content a total of 124 records were assessed for eligibility based on their titles and abstracts. This screening of 124 records led to the removal of 108 articles, leaving 16 reports that were sought for full-text retrieval and assessed for eligibility against the inclusion criteria. Ten reports were excluded for the following reasons: the population was not K-12 (*n* = 2), the intervention did not meet eligibility criteria (*n* = 2), or the study design was insufficient (e.g., non-RCT, quasi-experimental, or lacked a control group) (*n* = 6). In total, six studies fulfilled all inclusion criteria and were incorporated into the final synthesis.

### Data extraction

A structured data extraction form was used to collect relevant information from the 6 included studies (Page et al., 2021). The extracted data included: (1) study details (author, year), (2) study design, (3) participant demographics (sample size, age, grade level), (4) intervention details (VR technology, duration, activities), (5) primary outcome measures, and (6) key findings, including statistical results and effect sizes. One reviewer extracted the data, and a second reviewer cross-checked the extracted information for accuracy and completeness.

### Risk of bias assessment

Methodological quality was independently appraised by two reviewers (Author A and Author B) employing validated tools tailored to the study design. The six randomized controlled trials underwent evaluation using the Cochrane Risk of Bias 2 (RoB 2) framework (Sterne et al., 2019). Any discrepancies between reviewers were addressed through deliberation until agreement was achieved. The detailed risk-of-bias outcomes are presented in the Results section.

### Data synthesis

Due to substantial heterogeneity in intervention designs, participant characteristics, and outcome measures across the included studies, a meta-analysis was deemed inappropriate. Instead, a narrative synthesis was conducted following the Synthesis Without Meta-analysis (SWiM) guideline (Campbell et al., 2020). We grouped studies according to the type of comparison (primary contrast: VR vs. non-VR; secondary analysis: VR vs. VR) and learning domain. For each study, we narratively summarized the findings by reporting the direction of effect and the reported effect size (e.g., Cohen's *d*, partial eta squared) to describe the magnitude of the intervention's impact. We did not use vote-counting based on statistical significance to synthesize results.

Although several studies reported effect sizes, the small number of trials per outcome domain, inconsistent reporting of variance estimates, and differences in post-test timing and assessment instruments meant that any pooled quantitative estimate would have been statistically fragile and potentially misleading. Adopting the SWiM framework therefore allowed us to pre-specify grouping rules, summarize the direction and magnitude of effects in a transparent manner, and explicitly acknowledge heterogeneity without overstating the precision of the evidence.

## Results

### Characteristics of included studies

The key features of the six included studies are outlined in Table 1. All were published within the recent time frame of 2021–2025, reflecting a current emphasis in the field. Geographically, the studies were mainly concentrated in East Asia, with five originating from China (including four conducted in Taiwan region of China), one from South Korea, and another from the United States. Each of the selected studies adopted a rigorous experimental design, specifically a randomized controlled trial (RCT) methodology.

**Table 1:** Characteristics of included studies.

**Study**	**Study design**	**Participants (grade/age)**	**VR technology type**	**Language skills targeted**
Chang et al. (2024)	Randomized Controlled Trial (RCT)	Grades 2–3, 300 participants, Korean elementary schools	HMD-based system (VIVE Pro) with interactive controllers (VIVE Pro controllers, VIVE MAG P90 Gun) and a 360-degree stereoscopic screen display	English learning (action keywords, vocabulary, expressions)
Lai and Chen (2023)	Randomized Controlled Trial (RCT)	Grade 12, 17–18 years, 30 participants, China	Oculus Go, “Angels and Demigods” VR visual novel	Vocabulary acquisition and retention
Tai et al. (2022)	Randomized Controlled Trial (RCT)	Grade 7, 14–15 years, 49 participants, China	Samsung Gear VR, “Mondly” VR app	Vocabulary learning and retention
Tai and Chen (2021)	Randomized Controlled Trial (RCT)	Grade 7, 13–15 years, 72 participants, China	Samsung Gear VR, “Mondly” VR app	Listening comprehension and retention
Kaplan-Rakowski and Thrasher (2024)	Randomized Controlled Trial (RCT)	Grades 9–12 (High School), 13–18 years, 91 participants, USA	Meta Quest 2, “Immerse” platform	Vocabulary learning and retention
Guan et al. (2024)	Randomized Controlled Trial (RCT)	Grades 7–9 (Junior High), 12–15 years, 63 participants, China	HTC Vive Pro2	Writing performance and empathy

Participants in these studies covered a wide K-12 age range, from lower elementary (Grades 2–3) to junior high (Grade 7) and high school (Grades 9–12). Sample sizes varied considerably, from 30 to 300 students.

The interventions featured a diverse array of VR technologies. Hardware included both mobile VR systems like the Oculus Go and Samsung Gear VR, as well as more powerful standalone or PC-tethered head-mounted displays (HMDs) such as the Meta Quest 2 and HTC Vive Pro2. The learning content was delivered through various software, including commercial language learning platforms like “Mondly” and “Immerse”, and specific VR games or experiences like “Angels and Demigods”.

Notably, while one included study (Chang et al., 2024) utilized a 360-degree screen projection system, its core mechanism involved participants wearing VIVE Pro HMDs for motion tracking and using handheld controllers for direct interaction, thus aligning with our operational definition of immersive VR.

The primary focus of the interventions was on vocabulary acquisition and retention, which was the main outcome in four of the six studies. Other targeted language skills included listening comprehension and writing performance. One study also uniquely investigated the development of empathy alongside writing skills through a custom-designed empathetic VR approach.

### Effects of virtual reality on language learning outcomes

To address our primary research questions regarding the effectiveness of VR against non-VR instructional methods, we first report on the primary contrast, which includes four trials that compared an immersive VR group to a non-VR control group (e.g., video-watching, PC-based games, traditional instruction). Following this, we present a secondary analysis of two trials that compared different types of VR interventions (e.g., high-immersion vs. low-immersion VR; empathetic vs. standard VR) to explore within-modality design effects.

Across the included studies, the direction of effect in the comparisons consistently favored the VR interventions over control conditions, although not all findings reached statistical significance. The findings for each targeted skill, summarized in Table 2, are detailed below.

**Table 2:** Summary table: language skill outcomes.

**Study**	**Language skill**	**VR condition results**	**Control condition results**	**Effect direction**	**Effect size (95% CI)**
Chang et al. (2024)	English Learning (action keywords, vocabulary, expressions)	Significantly higher total scores, specifically in “Action Keywords” and “Vocab” sections.	Lower scores in total, “Action Keywords,” and “Vocab”.	VR > Control	/
Lai and Chen (2023)	Vocabulary (translation, recognition)	Significantly higher scores on the delayed translation posttest. No significant difference in recognition tests.	Lower scores on the delayed translation posttest.	VR > Control (for translation retention)	/
Tai et al. (2022)	Vocabulary (definition-supply, cloze)	Significantly higher scores on posttest and delayed posttest for definition-supply and on the delayed cloze test.	Lower scores on posttest and delayed posttest.	VR > Control	Immediate: η^2^ = 0.22; Delayed: η^2^ = 0.17
Tai and Chen (2021)	Listening comprehension	Significantly higher scores on the listening comprehension posttest and in the free recall of main ideas and details.	Lower scores on listening comprehension and free recall tasks.	VR group outperformed control on immediate comprehension (*d* = 0.50) and retention of main ideas (*d* = 1.15).	Immediate: *d* = 0.50; Retention: *d* = 1.15
Kaplan-Rakowski and Thrasher (2024)	Vocabulary (productive, receptive)	Marginally significantly higher scores on the delayed receptive posttest (*p* = 0.06). No other significant differences found.	Lower scores on the delayed receptive posttest.	VR > Control (approaching significance for receptive retention)	Retention: η^2^ = 0.08
Guan et al. (2024)	Writing Performance (ideas and content, word choice, voice)	Significantly higher overall writing scores, specifically in the dimensions of “ideas and content,” “word choice,” and “voice”.	Lower writing scores in the mentioned dimensions.	Empathetic VR group showed significantly higher overall writing scores compared to standard VR (η^2^ = 0.170).	η^2^ = 0.170

In the domain of listening comprehension, the single primary contrast study (Tai and Chen, 2021) found that the VR group demonstrated a moderate advantage over the control group in immediate comprehension (Cohen's *d* = 0.50) and a large advantage in the retention of idea units (*d* = 1.15). These effect sizes suggest a practically meaningful benefit for the immersive VR intervention, particularly for long-term recall.

For writing performance, Guan et al. (2024) found that an empathetic VR approach led to significantly better overall writing scores compared to a standard VR approach (η^2^ = 0.170, indicating a large effect). The improvements were most pronounced in the qualitative dimensions of writing, such as “ideas and content,” “word choice,” and “voice,” suggesting that VR can enhance deeper aspects of writing proficiency.

Vocabulary acquisition and retention were the most frequently assessed outcomes; Of the four studies that assessed vocabulary, the results were mixed regarding immediate learning gains. Two studies reported statistically significant advantages for immersive VR over control conditions, while the other two found no significant difference. For instance, Chang et al. (2024) observed higher vocabulary scores in the VR group than in the traditional-instruction control (*p* < 0.01). Tai et al. (2022) likewise found the VR group outperformed a video-viewing control on both immediate (η^2^ = 0.22, indicating a large effect) and delayed (η^2^ = 0.17, indicating a large effect) vocabulary tests. Lai and Chen (2023) further underscored VR's benefits for longer-term retention: scores were comparable on the immediate post-test, but the VR group exceeded the non-VR comparison (PC) group on a delayed translation test assessing productive vocabulary knowledge (*p* = 0.004). Study four (Kaplan-Rakowski and Thrasher, 2024) purported a less definitive outcome since no statistically significant difference materialized between high-immersion VR (HiVR) and low-immersion VR (LiVR) during a measure of immediate tests. For long-term retention of receptive vocabulary, the analysis revealed a small positive effect in favor of the HiVR group (η^2^ = 0.08), however, this result was not statistically significant (*p* = 0.06), indicating that a true difference cannot be concluded with confidence from this data alone.

Across the targeted skills, the included trials consistently demonstrated a positive direction of effect for VR interventions over control conditions.

### VR technology features and learning effectiveness

Beyond the general finding that VR is effective, this review identified several key technological features and pedagogical approaches that appear to underpin its success in K-12 language education. These features primarily relate to the levels of immersion, interactivity, and the contextual integration of content.

A central feature of the interventions was the use of immersion. Studies employing high-immersion VR technologies consistently reported positive learning outcomes. The sense of presence and embodied interaction within these immersive environments were frequently cited as mechanisms driving these improvements (Ratcliffe and Tokarchuk, 2020). However, the relationship is not simply that higher immersion is always better. The study by Kaplan-Rakowski and Thrasher (2024) provided a more nuanced perspective, finding no significant difference between high-immersion (HiVR) and low-immersion VR (LiVR) in immediate vocabulary gains, but suggesting a potential advantage for HiVR in long-term retention. This shows that both levels of immersion can work, but high immersion may have special benefits for memory consolidation and deeper cognitive processing.

Interactivity was a consistent correlate of stronger outcomes. Interventions that incorporated branched dialogue with virtual agents, manipulable objects, and real-time feedback tended to yield higher engagement and better learning performance (Parong and Mayer, 2018). This aligns with constructivist learning theories, which posit that learners build knowledge most effectively through active engagement and discovery a process that highly interactive VR environments are well-suited to facilitate. Several studies also combined gamification and collaborative tasks, further enhancing both engagement and learning outcomes.

Finally, effects were strongest when VR content was authentic and task-relevant. By situating learners in ecologically valid scenarios (e.g., navigating a police station or a shopping mall), VR rendered target-language use purposeful. Such designs typically leveraged multimodal input—visual, auditory, and kinesthetic supporting embodied processing and deeper comprehension (Makransky and Petersen, 2021). Some interventions taught more than just language skills; they also included cultural content and even activities to help students understand how other people feel. This made the learning process more complete and useful.

### Risk of bias

**Figure 2:**
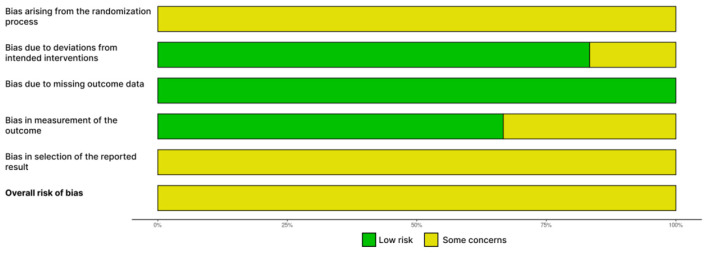
Risk of bias summary: review authors' judgements about each risk of bias item presented as percentages across all included studies.

**Figure 3:**
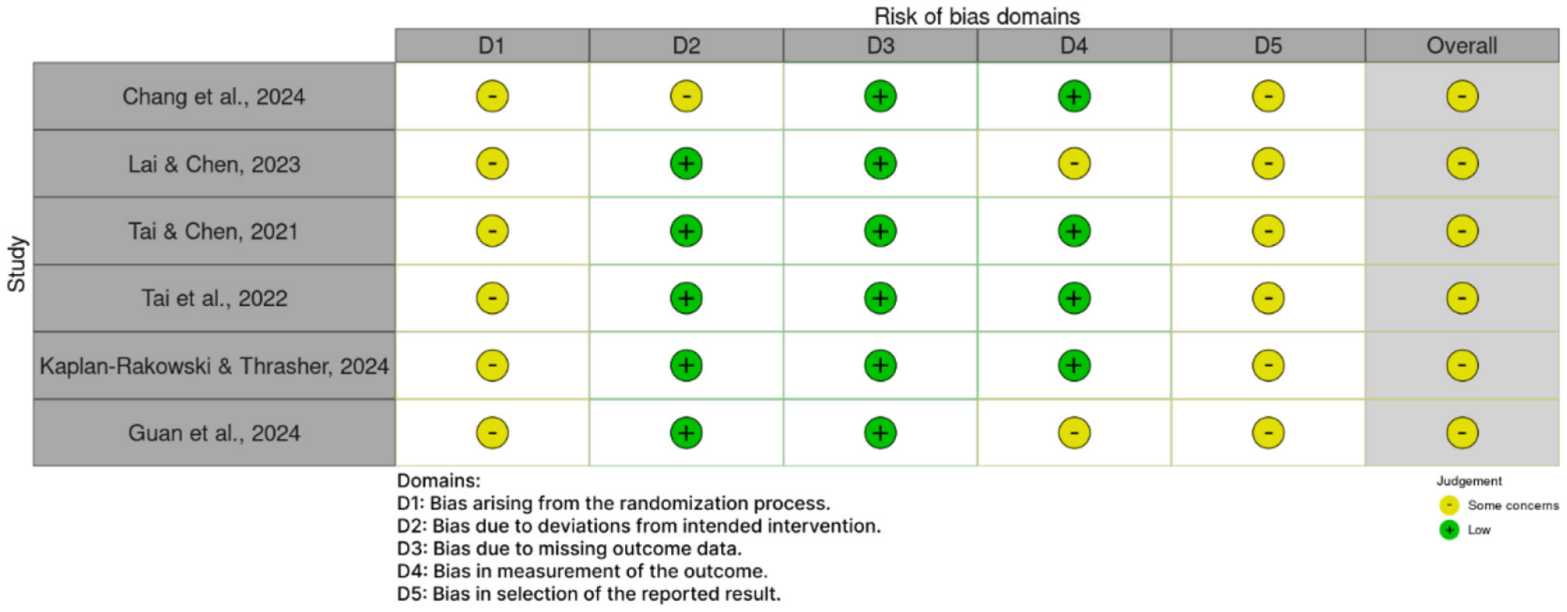
Risk of bias traffic light plot: review authors' judgements about each risk of bias item for each included study.

The methodological quality of the six included trials was assessed using the Cochrane Risk of Bias 2 (RoB 2) tool (Sterne et al., 2019). Overall, all six trials were rated as having “Some concerns” for bias. A summary of the risk of bias assessments across all studies is presented in Figure 2, with a detailed study-by-study breakdown provided in Figure 3.

As shown in Figure 2, consistent concerns were identified across two primary domains: D1 (bias arising from the randomization process) and D5 (bias in selection of the reported result), where 100% of studies were rated as having “some concerns.” In contrast, the risk of bias was generally low for D3 (bias due to missing outcome data), where all studies were rated as low risk.

The “Some concerns” ratings in Domain one were primarily due to insufficient reporting on allocation concealment or the method of random sequence generation. Similarly, concerns in Domain five arose because most studies did not have a prospectively registered protocol, making it impossible to rule out the possibility of selective outcome reporting.

### Secondary analysis: comparing different VR designs

Two studies moved beyond the VR vs. non-VR paradigm to investigate the effects of different VR design features. Kaplan-Rakowski and Thrasher (2024) compared a high-immersion VR (HiVR) condition with a low-immersion VR (LiVR) condition for vocabulary learning. They found no significant difference in immediate tests, but reported a trend favoring the HiVR group for long-term retention of receptive vocabulary (*p* = 0.06). Similarly, (Guan et al., 2024) compared an empathetic VR approach where students were placed in narrative, perspective-taking scenarios designed to evoke emotional engagement to a standard VR approach that delivered the same writing task in a more neutral, task-focused environment. Their findings indicated that the empathetic VR design led to significantly higher overall writing scores, particularly in qualitative dimensions like “ideas and content” and “word choice”.

## Discussion

### Principal findings and interpretations

The principal finding from our primary contrast analysis, which synthesized four RCTs comparing immersive VR against non-VR conditions, is that VR demonstrates a promising, albeit inconsistent, advantage. Given that only six RCTs met the inclusion criteria and that they differ markedly in age groups, VR hardware, target skills, and outcome measures, these findings should be regarded as preliminary signals rather than firm, generalizable conclusions. The most consistent benefit appeared in long-term knowledge retention, particularly for vocabulary, where VR groups consistently outperformed controls in delayed post-tests (Lai and Chen, 2023; Tai et al., 2022). In contrast, the evidence for immediate learning gains was inconclusive, with half of the primary contrast studies showing a significant benefit and half showing no difference. For listening comprehension, the single primary contrast study found a moderate-to-large positive effect for VR (Tai and Chen, 2021).

Furthermore, the secondary analysis of two studies comparing different VR designs provides critical insights. The findings suggest that specific design features, such as the level of immersion or the integration of empathetic narratives, are key variables that can significantly influence learning outcomes in domains like vocabulary retention (Kaplan-Rakowski and Thrasher, 2024) and writing performance (Guan et al., 2024). This underscores that “VR” is not a monolithic treatment; its effectiveness is highly dependent on its specific design and pedagogical implementation.

The technological features of the interventions appear to be central to their success. Immersion and interactivity were consistently highlighted as critical components that foster student engagement and positive learning outcomes (Huang et al., 2021; Kaplan-Rakowski and Thrasher, 2025). However, the review also suggests that the relationship between the level of immersion and learning effectiveness is not linear. The finding that high-immersion VR did not significantly outperform low-immersion VR on immediate tests, but showed a potential advantage for long-term retention, is particularly salient (Cadet and Chainay, 2020; Kaplan-Rakowski and Thrasher, 2025). This indicates that while various levels of immersion can be effective, high immersion might offer unique benefits for long-term memory, a crucial area for future investigation (Kaplan-Rakowski and Thrasher, 2025; Xie et al., 2025).

However, it is crucial to interpret these positive trends with significant caution due to the extreme heterogeneity across the included studies. The wide range of participant ages, from lower elementary to high school, and the disparity in VR technology, from mobile VR to high-end PC-tethered systems, prevent a monolithic conclusion about VR's effectiveness. For instance, the significant effects observed in studies using high-fidelity VR with older students (Guan et al., 2024; Kaplan-Rakowski and Thrasher, 2024) may not be generalizable to contexts using simpler technology with younger children (Chang et al., 2024). Therefore, a key finding of this review is not simply that VR has potential, but that the current K-12 RCT evidence base is too fragmented to draw firm conclusions, highlighting the urgent need for future research to investigate these moderating variables.

This heterogeneity in participant age and technology likely acts as a significant moderating variable (Hite et al., 2019). For instance, the pedagogical design for younger elementary students may require more structured guidance and gamified elements to maintain engagement, whereas high school students might benefit more from complex, open-ended exploratory environments. The cognitive load imposed by different VR systems could also explain varied outcomes (Makransky and Petersen, 2021; Parong and Mayer, 2018).

High-end, tethered VR systems offer greater immersion and interactivity, which may enhance learning and retention, but could also overwhelm younger learners (Huang et al., 2021). Conversely, simpler mobile VR systems are more accessible but may not provide the same level of presence needed to foster deep learning (Wilkinson et al., 2021). Future research should therefore not only compare VR to non-VR conditions but also conduct head-to-head comparisons of different VR designs and technologies to isolate these influential factors.

### Strengths and limitations of the review

This review possesses several methodological advantages, such as an extensive literature search conducted across five major academic databases, alignment with PRISMA reporting standards, and the application of established tools (RoB 2) for assessing potential bias. However, a number of limitations should also be considered. First, the evidence base consists of only six RCTs that are highly heterogeneous in terms of participant age, intervention content, VR hardware, and outcome measures. This small and varied corpus precluded a meaningful meta-analysis and prevented formal investigation of moderators or publication bias, which further constrains the generalizability of our conclusions. Specifically, the inclusion of one study using a 360-degree projection system alongside HMDs, while justified by its interactive nature, introduces technological heterogeneity that may limit the generalizability of the pooled findings. A sensitivity analysis was considered to assess the stability of our findings. Given the narrative nature of this review, this analysis remained qualitative. If the study by Chang et al. (2024) were to be excluded, the overall conclusion of this review would not substantially change. The evidence for vocabulary and listening would remain mixed, and the most consistent finding would still be the potential benefit of VR for long-term retention, as supported by other included studies. However, the removal would increase the technological homogeneity of the evidence base, thereby strengthening the internal validity of our synthesis regarding HMD-based VR. Second, all six studies were rated as having “Some concerns” with respect to bias, particularly in domains associated with randomization procedures and selective reporting of results. As a result, the findings should be interpreted with appropriate caution. Third, the geographic concentration of the studies in East Asia may constrain the extent to which these findings are applicable to other educational and cultural settings. More specifically, the school systems represented in these East Asian contexts often differ from those in other regions in terms of curriculum structure, examination pressure, and access to immersive technologies. As such, the positive effects observed in Chinese mainland, Taiwan region of China, and South Korean classrooms should be interpreted as most securely applicable to East-Asian-style K−12 systems, rather than being generalized to all global contexts. In addition, none of the included RCTs evaluated speaking or oral production as a primary outcome, even though immersive VR is theoretically well suited to support embodied, interactive communication. This omission means that our synthesis can say little about perhaps the most promising skill domain L2 speaking and underscores the need for future trials that incorporate rigorous, VR-specific speaking measures. Implications for practice and avenues for future research are discussed accordingly.

The protocol for this review was preregistered with the Open Science Framework. We report one main deviation from the registered protocol: the data synthesis approach was updated from a planned vote-counting method to a narrative synthesis following the SWiM guideline. This change was made *post-hoc* to adopt a more rigorous and informative synthesis methodology. Consequently, the structuring of the results into a primary and secondary analysis also represents a deviation aimed at improving clarity.

### Implications for practice and future research

The results of this review have significant ramifications for both practical application and subsequent research endeavors. For practitioners, this review provides crucial, RCT-based evidence to guide the integration of VR into K-12 language curricula, emphasizing the development of interactive, contextually rich experiences that enhance long-term knowledge retention (Essoe et al., 2022; Ng et al., 2023; Xie et al., 2025). This review highlights the need for more methodologically rigorous randomized controlled trials (RCTs) with low risk of bias, larger samples, and broader geographic coverage. Future work should include head-to-head comparisons of VR design features (e.g., immersion level, interactivity type) and extend evaluation to understudied skills such as speaking and pragmatic competence. Longer-term follow-up is also essential to test the durability of effects and substantiate VR's benefits for retention. Notably, none of the included RCTs measured oral production or speaking skills as a primary outcome. This represents a significant gap in the current high-quality evidence base and should be a priority for future research in this domain.

## Conclusion

Across a small and heterogeneous set of recent RCTs, immersive VR shows promising effects—especially for long-term retention. However, the evidence for immediate learning gains is inconclusive and varies by domain. A more critical finding is the profound heterogeneity and methodological concerns (all included studies rated as having “some concerns” for bias) within the current evidence base, which preclude any single, overarching conclusion about VR's effectiveness. The significant variation in participant age, intervention design, and technology type complicates any single, overarching conclusion and underscores that the effectiveness of VR is likely context-dependent (Dhimolea et al., 2022; Dooly et al., 2023; Frolli et al., 2024; Li, 2023). Across the included trials, VR interventions tended to yield better outcomes than control conditions for vocabulary acquisition, listening comprehension, and writing proficiency (Frolli et al., 2024; Li et al., 2021). The most consistently reported advantage of VR is improved long-term retention; its immersive and interactive properties likely support deeper encoding and more durable learning (Pellas et al., 2021). While the findings are encouraging, the evidence base remains nascent and constrained by methodological limitations, warranting cautious interpretation. Therefore, while VR holds considerable potential to transform language education, further high-quality research is necessary to substantiate these findings and develop clear guidelines for its optimal implementation in diverse educational settings.

Crucially, as all six included trials were assessed as having 'some concerns' regarding risk of bias, these encouraging findings must be viewed as preliminary and require validation through more methodologically robust research.

## Data Availability

Publicly available datasets were analyzed in this study. The pre-registered protocol for this systematic review is available on the Open Science Framework (OSF) at the following repository: https://osf.io/kp78r/?view_only=7d2c8fe674d9480ca6bc45d6fe751fd5. The detailed data extraction sheets, full Risk of Bias 2 (RoB 2) assessment forms, and the PRISMA checklist are not publicly archived but are available from the corresponding author upon reasonable request.
